# Current Immunotherapeutic Approaches in Pancreatic Cancer

**DOI:** 10.1155/2011/267539

**Published:** 2011-09-14

**Authors:** Shigeo Koido, Sadamu Homma, Akitaka Takahara, Yoshihisa Namiki, Shintaro Tsukinaga, Jimi Mitobe, Shunichi Odahara, Toyokazu Yukawa, Hiroshi Matsudaira, Keisuke Nagatsuma, Kan Uchiyama, Kenichi Satoh, Masaki Ito, Hideo Komita, Hiroshi Arakawa, Toshifumi Ohkusa, Jianlin Gong, Hisao Tajiri

**Affiliations:** ^1^Division of Gastroenterology and Hepatology, Department of Internal Medicine, The Jikei University School of Medicine, Tokyo 105-8461, Japan; ^2^Institute of Clinical Medicine and Research, The Jikei University School of Medicine, Tokyo 105-8461, Japan; ^3^Department of Oncology, Institute of DNA Medicine, The Jikei University School of Medicine, Tokyo 105-8461, Japan; ^4^Department of Medicine, Boston University School of Medicine, Boston, MA 02118, USA

## Abstract

Pancreatic cancer is a highly aggressive and notoriously difficult to treat. As the vast majority of patients are diagnosed at advanced stage of the disease, only a small population is curative by surgical resection. Although gemcitabine-based chemotherapy is typically offered as standard of care, most patients do not survive longer than 6 months. Thus, new therapeutic approaches are needed. Pancreatic cancer cells that develop gemcitabine resistance would still be suitable targets for immunotherapy. Therefore, one promising treatment approach may be immunotherapy that is designed to target pancreatic-cancer-associated antigens. In this paper, we detail recent work in immunotherapy and the advances in concept of combination therapy of immunotherapy and chemotherapy. We offer our perspective on how to increase the clinical efficacy of immunotherapies for pancreatic cancer.

## 1. Pancreatic Cancer

Pancreatic tumors usually display a ductal, an acinar, or an endocrine differentiation. The majority (approximately 95%) of pancreatic tumors arise from the exocrine component of the pancreas, and of these the significantly most common is ductal adenocarcinoma [1]. Pancreatic adenocarcinoma that is the fifth leading cause of cancer death worldwide is a lethal disease with an overall 5-year survival of only 6% [1]. Moreover, for locally advanced cancer patients, the life expectancy is about 6-8 months [1]. No adequate therapy for pancreatic cancer has yet been found, and most of patients diagnosed annually die within a year of diagnosis. Despite recent improvements in diagnostic techniques, pancreatic cancer is diagnosed at an advanced stage in most patients. Therefore, surgical resection (pancreaticoduodenectomy) can be performed in only a small number of patients [2]. Even after resection, recurrence occurs in the majority of the patients, leading to a median survival of about 18 months after resection. Although adjuvant treatment with both chemotherapy and radiation therapy was investigated, which demonstrated improvements in disease-free survival and overall survival rates [3], new therapeutic approaches are still needed.

## 2. Cytotoxic Chemotherapeutic Agents

Gemcitabine (2′2′-difluorodeoxycytidine) is a chemotherapeutic drug that has become the standard treatment for advanced disease after showing superiority over 5-fluorouracil (5-FU), while chemoradiation plus systemic chemotherapy is also still widely used [4]. Therefore, gemcitabine was established as the standard first line treatment for patients with advanced disease. Gemcitabine is a nucleoside analogue that exerts its antitumor activity via multiple mechanisms of action. These include (1) incorporation of gemcitabine into replicating DNA, which inhibits DNA replication and cell growth, (2) masked DNA chain termination, and (3) several self-potentiation mechanisms that serve to increase intracellular levels of the active compound [5]. It thus halts DNA synthesis and is invisible to DNA repair systems, leading the cells into the apoptotic pathway. However, most patients treated with gemcitabine do not survive longer than 6 months, as the tumor cells are naturally resistant to current chemotherapy. Subsequent trials aimed at improving survival have combined gemcitabine with various cytotoxic (platinums, fluoropyrimidines, or topoisomerase inhibitors) [6–10], or biological agents (tipifarnib [11], marimastat [12], or cetuximab [13]). However, the addition of the cytotoxic agents to gemcitabine did not lead to a statistically significant improvement in overall survival (OS) in patients with advanced pancreatic cancer [14–17]. 

## 3. Biological Agents

Some therapies based on mechanisms that target specific biologic pathways of tumors have commonly been referred to as “targeted therapy.” While traditional cytotoxic drugs also target specific cellular process, the newer generation of agents is set apart by their targeting of a pathway or molecular that derives the growth, speed, survival, or maintenance of tumor cells specially. There is a sound rationale for combining a human epidermal growth factor receptor type 1 (HER1/EGFR) inhibitor and gemcitabine in pancreatic cancer. Erlotinib (Taraceva, Genentech, South San Francisco) is a small molecule HER1/EGFR tyrosine kinase inhibitor. The human HER1/EGFR is overexpressed in many pancreatic tumors and is associated with more aggressive disease and poorer outcome [18, 19]. Blocking HER1/EGFR tyrosine kinase signaling improves the anticancer effects of gemcitabine [20]. Indeed, the combination of gemcitabine plus erlotinib significantly improved OS compared with gemcitabine alone [17]. This combination therapy first provided proof of principle of targeting HER1/EGFR in pancreatic cancer and showed erlotinib-improved survival when used concurrently with gemcitabine. Therefore, the US Food and Drug Administration (FDA) recently approved erlotinib for use in the first-line setting of advanced pancreatic cancer in combination with gemcitabine. However, this survival benefit was small, and the combination therapy increased the cost; therefore, erlotinib has not yet been widely incorporated into standard treatment protocols. Another study evaluating EGFR as a target in pancreatic cancer, using the monoclonal antibody cetuximab, has been completed. In patients with advanced pancreas cancer, cetuximab did not improve the outcome compared with patients treated with gemcitabine alone [13]. Moreover, studies evaluating monoclonal antibodies to vascular endothelial growth factor (VEGF) and using combinations of targeted agents in patients with advanced pancreatic cancer are underway.

## 4. Immunotherapy

**Figure 1:**
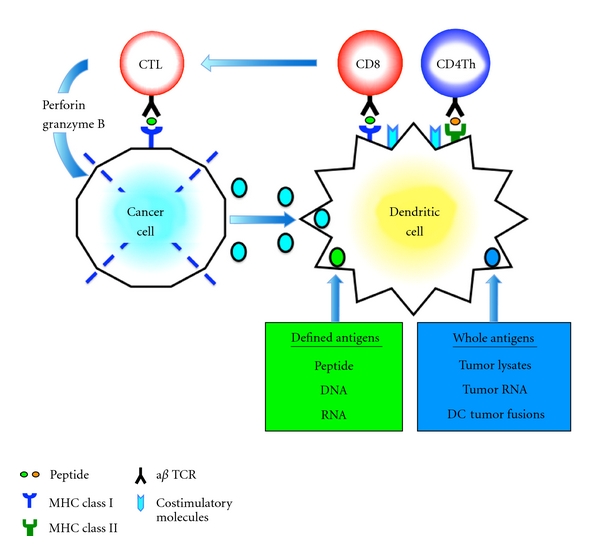
CTL induction by DCs. Antigens are taken up and degraded into peptide fragments by antigen-presenting cells, such as immature DCs. DCs process tumor-derived peptides and MHC class I peptides derived from DCs. They form MHC class I-peptide complexes, in the endoplasmic reticulum, which are transported to the surface of DCs and presented to CD8+ T cells. DCs also synthesize MHC class II peptides in the endoplasmic reticulum, which are transported to the cytoplasm where MHC class II-peptide complexes are assembled with tumor-derived peptides and presented to CD4+ T cells. The CD4+ T cells produce increased amounts of IL-2, which drives CD8+ T-cell proliferation. CD8+ T cells then become CTL, which can destroy cancer cells through effector molecules such as granzyme B and perforin.

**Figure 2:**
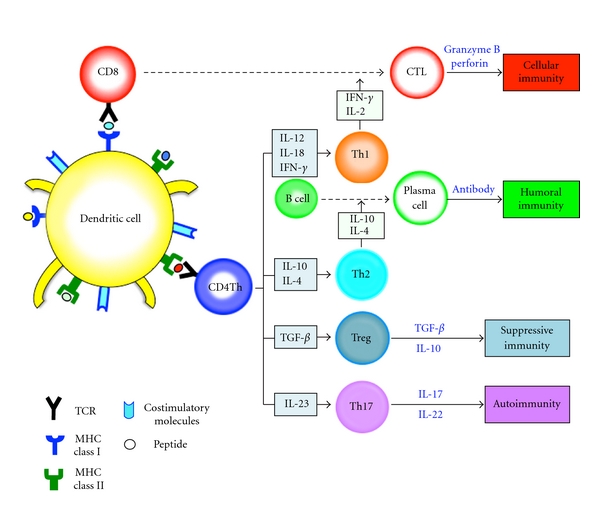
Immune homeostasis. Upon TCR-mediated cell activation, naive CD4 T cells can differentiate into four major lineages, Th1, Th2, Th17, and Treg cells that participate in different types of immune responses. The Th1 cells produce IFN-*γ* and IL-2, resulting in induction of CD8+ CTLs. Th2 cells secrete IL-4 and IL-10. The Th2 response is associated with the humoral, antibody-based antitumor response. Th17 cells secrete IL-17 and IL-22, eliciting tissue inflammation implicated in autoimmunity. Treg cells that secrete TGF-*β* and IL-10 suppress effector Th1 or Th2 cells.

The aim of antitumor immunotherapy is to induce efficient cytotoxic T lymphocyte (CTL) responses against pancreatic cancer cell. Dendritic cells (DCs) are powerful antigen-presenting cells (APCs) that play a pivotal role in the initiation, programming, and regulation of tumor-specific immune responses [21, 22]. DCs can process endogenously synthesized antigens or exogenous antigens into antigenic peptides, presented to the cell surface as MHC class I-peptide complexes, and recognized by the *αβ* T cell receptor (TCR) in CD8+ T cells [23]. In contrast, exogenous antigens are captured and delivered to the compartments of the endosome/lysosome, where they are degraded to antigenic peptides by proteases and peptidases, which are complexed with MHC class II and recognized by the *αβ* TCR in CD4+ T cells [23–25]. The *αβ* TCR in CD8+ CTL can recognize MHC class I-peptide complexes on cancer cells and destroy cancer cells through effector molecules such as granzyme B and perforin (Figure 1) [26, 27]. Upon TCR-mediated cell activation, naive CD4+ T cells can differentiate into at least four major lineages, Th1, Th2, Th17, and regulatory T (Treg) cells all of which participate in different types of immune responses (Figure 2) [28]. The Th1 cells produce interferon (IFN)-*γ* along with proinflammatory cytokines, such as tumor necrosis factor (TNF)-*α* and TNF-*β*, to activate DCs, which can regulate the survival and persistence of CD8+ CTLs as memory cells [24, 29]. Th2 cells secrete interleukin (IL)-4 and IL-10 [24, 29]. The Th2 response is often associated with the humoral, antibody-based antitumor response [30, 31]. Th17 cells secrete IL-17 and IL-22, eliciting tissue inflammation implicated in autoimmunity [32–34]. There are increasing evidences that cancer cells-derived soluble factors promote the induction of tolerance through the generation of CD4+ *α* chain of IL-2R (CD25)+ forkhead box P3 (Foxp3)+ Treg subset, which is linked to compromised antitumor immune responses [35]. 

**Figure 3:**
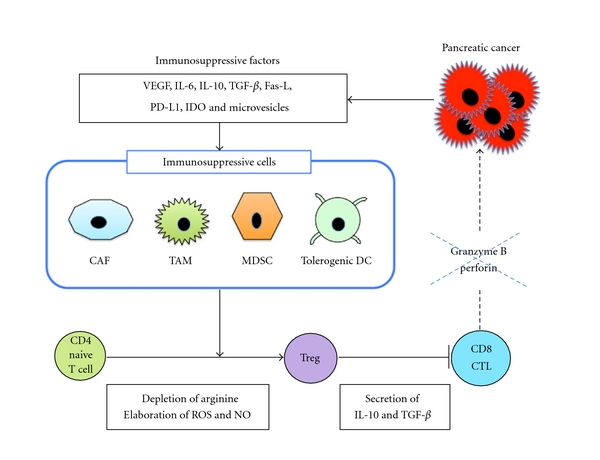
Pancreatic cancers induce an immunosuppressive tumor microenvironment. Pancreatic cancer cells secrete various immunosuppressive factors such as VEGF, IL-6, IL-10, TGF-**β**, Fas-L, IDO, PD-L1, and microvesicles, all of which promote the accumulation of TAM, MDSC, or tolerogenic DC. These immunosuppressive cells inhibit antitumor immunity by various mechanisms, including depletion of arginine and elaboration of ROS and NO. An immunosuppressive tumor microenvironment induced by pancreatic cancers suppresses CD8+ CTL function through secretion of IL-10 and TGF-*β* from Treg cells. All contribute to pancreatic cancer-induced immune suppression.

The field of cancer immunotherapy is currently in an active state of preclinical and clinical investigations. The development of new treatment modalities, including specific immunotherapy, is of great importance in the treatment of pancreatic cancer. In support of the immunotherapy approach are the findings that pancreatic cancer cells express TAAs such as Wilms' tumor gene 1 (WT1) (75%) [36], mucin 1 (MUC1) (over 85%) [37], human telomerase reverse transcriptase (hTERT) (88%) [38], mutated K-RAS (73%) [38, 39], survivin (77%) [40], carcinoembryonic antigen (CEA) (over 90%) [41], HER-2/neu (61.2%) [42], or p53 (67%) [43] as potential targets for immunotherapy. Immunotherapies aim to recruit and activate T cells that recognize TAAs-specific antigens. Moreover, pancreatic cancer cells themselves actively contribute to immune suppression through production of immune suppressive cytokines (e.g., TGF-*β*, IL-10, and IL-6) and by expressing surface molecules that mediate immune suppression (e.g., vascular endothelial growth factor (VEGF), Fas ligand (Fas-L), programmed death-1 ligand (PD-L1) and indolamine-2, and 3-dioxygenase (IDO)) [44]. In addition, the environment in pancreatic cancer is consisted of not only cancer cells but also immune suppressive cells such as cancer-associated fibroblasts (CAFs), tolerogenic DCs, myeloid-derived suppressor cells (MDSCs), immunosuppressive tumor-associated macrophages (TAMs), and Treg cells [44] (Figure 3). As a result, immunosuppressive cells inhibit antitumor immunity by various mechanisms, including depletion of arginine and elaboration of reactive oxygen species (ROS) and nitrogen oxide (NO) [44]. Finally, an immunosuppressive tumor microenvironment induced by pancreatic cancers suppresses CD8+ CTL function through secretion of IL-10 and TGF-*β* from Treg cells [45, 46]. The accumulation of these immune suppressive cells in pancreatic cancer might be closely related to the extent of disease and correlated well with disease stage. Therefore, immunotherapies that struggle against pancreatic cancer cells with antigen-specific CTLs as well as depletion of Treg cells may tip the balance in favor of immunostimulation. Currently, the field of cancer immunotherapy using peptide- or cell- (DC or whole tumor cell)-based approaches is in an active state of preclinical and clinical investigations.

## 5. Peptide Vaccines

TCR engagement by peptide/MHC constitutes the main signal for the activation of naive CD4+ and CD8+ T cells. Although CD8+ naive T cells recognize peptides derived from TAAs bound by MHC class I molecules, it is not sufficient to initiate a productive generation of antigen-specific CTLs. Full induction of CTLs requires additional signals driven by costimulatory molecules on DCs. CD8+ CTLs can respond to TAAs-derived peptides presented in the context of MHC class I molecules on tumor cells. Therefore, many investigators have tried to identify MHC class I-binding peptides that could be utilized to develop tumor vaccines for treatment of cancer patients. Peptide-based cancer vaccines are preparations made from antigenic protein fragments (called epitopes) that represent the minimal immunogenic region of antigens [47, 48]. The increased understanding of antigen recognition at molecular level has resulted in the development of rationally designed peptide vaccines. Indeed, the peptide-based cancer vaccines for pancreatic cancer have undergone phase I/II clinical trials [49, 50]. The major advantages of peptide vaccines are that they are simple, safe, stable, and economical. Induction of CTLs need peptides derived from TAAs to be presented on the surface of APCs such as DCs in the context of HLA molecules. However, several obstacles limit the widespread usefulness of peptide vaccines. The drawback of this strategy comes from numerous factors: (i) a limited number of known synthesized short peptides cannot be available in many HLA molecules [51–53], (ii) CD8+ CTLs may be ineffective in reacting with pancreatic cancer cells downregulated by certain tumor antigens and MHC class I molecules, which may appear during the course of tumor progression [22], (iii) impaired function of APCs in patients with advanced pancreatic cancer [54, 55], and (iv) MDSCs or Treg cells in tumor environment produce immunosuppressive cytokines such as IL-10 and TGF-*β* [26]. 

Vaccination with synthetic peptides, particularly MHC class I-binding epitopes, has been performed in pancreatic cancer (Table 1). In a phase I/II trials, vaccination for the patients with advanced pancreatic cancer using mutant K-ras [39, 56, 57], MUC1 [58, 59], or telomerase [60] peptides was significantly associated with immune responses. Gjertsen et al. [56] first reported mutant K-ras peptide vaccines for pancreatic cancer. Since native epitopes have relatively low immunogenicity, granulocyte-macrophage colony-stimulating factor (GM-CSF) was applied to achieve efficient vaccination in the study. Among 48 patients with pancreatic cancer (10 surgically resected and 38 with advanced disease), vaccination of mutant K-ras peptides in combination with GM-CSF resulted in immune responses and prolonged survival. Moreover, another group also reported that vaccination of 24 patients with resected pancreatic cancer with K-ras peptide in combination with GM-CSF proved to be safe without tumor regression [57]. In an MUC1 peptide vaccine, vaccination of 16 patients with resected or locally advanced pancreatic cancer with 100 mer MUC1 peptide and SB-AS2 adjuvant resulted in low but detectable MUC1-specific immune responses in some patients [59]. Moreover, vaccination with 100 mer MUC1 peptide and incomplete Freund's adjuvant resulted in increased circulating anti-MUC1 IgG antibody in some patients [58]. In addition, augmented immune responses and prolonged survival were observed following vaccination of advanced pancreatic cancer patients with telomerase peptide and GM-CSF [60]. Recent protocols using personalized peptides demonstrated frequent induction of tumor reactive T cells [61]. In this regimen, prevaccination peripheral blood mononuclear cells (PBMCs) were screened for their reactivity in vitro to each peptide in patients, and only the reactive peptides were vaccinated to 11 patients with advanced pancreatic cancer. In the personalized peptide vaccines, augmented immune responses to at least one of peptides used for vaccination were observed in the postvaccination PBMCs [62]. In these all peptide vaccines, only a limited success has occurred in clinical trials. The short peptide can be loaded exogenously in MHC class I molecules and presented by DCs within a few days after injection to the patients. Moreover, the short peptide vaccines are not immunogenic enough. The more attractive peptide-based vaccines may be synthetic long peptides to induce antigen-specific polyclonal CD4+ and CD8+ T cells [63]. As long synthetic peptides are not able to bind directly on MHC class I or II molecules on DCs, they need to be taken up, processed, and presented by DCs. The long peptide vaccines can present MHC class I- and II-restricted epitopes long time, thus eliciting both CD4- and CD8-mediated immune recognition [64]. Peptide vaccines aimed at the treatment of established cancer may require long-lived presentation of epitopes by MHC class I and II molecules on appropriately activated DCs. Such presentation is essential for induction of robust therapeutic T-cell responses.

In a phase I study using long synthetic mutant ras peptides, Weden et al. [65] treated 23 patients who were vaccinated after surgical resection for pancreatic cancer. Long-term immunological memory responses to the vaccines were observed. Strikingly, 10-year survival was 20% (four patients out of 20 evaluable) versus zero (0/87) in a cohort of nonvaccinated patient treated in the same period. Cancer vaccines for pancreatic cancer may be tested to prevent recurrence and metastasis after surgical resection. Furthermore, peptide vaccines to boost immune responses in combination with chemotherapy to overcome robust cancers may be the key elements for the development of therapeutic cancer vaccines. Indeed, Wobser et al. [40] reported a case of complete remission (CR) of liver metastasis of pancreatic cancer refractory to gemcitabine chemotherapy under vaccination with a survivin peptide.

## 6. Whole Tumor Cell Vaccines

Despite the identification of peptides, autologous whole tumor cells remain a potent vehicle for generating antitumor immunity. This is because tumor cells express all relevant candidate TAAs, including both known and unidentified. In the clinical setting, an autologous whole tumor cell vaccine depends on the availability of adequate numbers of tumor cells. As only 10–15% of pancreatic cancer patients diagnosed are eligible for surgical, autologous pancreatic cancer cells may not be provided in most of the patients. Moreover, even if the patients are treated by surgical resection, it is difficult to prepare sufficient numbers of tumor cells due to the length of culture time and potential contamination of bacteria and fungus [55, 66]. To circumvent this problem, allogeneic tumor cell lines may be used instead of autologous tumor cells [66]. This strategy has numerous advantages: (i) allogeneic tumor cell lines are well characterized as TAA source, (ii) specific TAAs do not need to be identified for vaccination, (iii) allogeneic tumor cell lines, which shared with TAAs, can grow well in vitro; thus, there is no limiting factor for preparation of tumor cells, (iv) it is not necessary to determine HLA typing of patients and allogeneic tumor cells, because autologous DCs can process and present multiple TAAs from allogeneic tumor cells owing to cross-presentation in the context of appropriate MHC class I and II alleles, and (v) polyclonal antigen-specific CD4+ and CD8+ T cells can be generated, which may protect against tumor escape variants. While currently explored allogeneic approaches in whole tumor cell-based vaccination procedures represent an improvement in terms of standardization over their autologous counterparts, they nevertheless entail the culture of large batches of cells under good manufacturing practice (GMP) grade conditions. One of major challenges to develop an allogeneic tumor cell-based vaccine strategy is to overcome the potential hazards of fetal calf serum (FCS) that limit safety in clinical trials [55]. Optimization of these in vitro culture methodologies is required. 

In clinical trials, allogeneic whole tumor cells (melanoma, prostate, and pancreatic cancer), transduced with GM-CSF, have been applied clinically and shown to induce antitumor immunity [67–69]. In this trial, whole allogeneic tumor cells were genetically modified to secrete the immune stimulatory cytokine, GM-CSF, and then irradiated to prevent further cell division. GM-CSF is now recognized to be the crucial growth and differentiation factor for DCs. Therefore, this approach is based on the concept that GM-CSF is required at the site of the tumor to effectively prime TAAs-specific immunity. Allogeneic GM-CSF-secreting pancreatic cancer vaccine was conducted (Table 2). The vaccines induced systemic antitumor immunity against autologous pancreatic cancer cells [67]. The same group [70] administrated GM-CSF-secreting allogeneic pancreatic cancer cells in sequence with cyclophosphamide in patients with advanced pancreatic cancer. The approach showed minimal treatment-related toxicity and mesothelin-specific T-cell responses. Moreover, combination of the vaccine and cyclophosphamide resulted in median survival in a gemcitabine-resistant population similar to chemotherapy alone. It was also reported that combination of the vaccines and chemoradiation demonstrated an overall survival that compares favorably with published data for resected pancreas cancer [69].

## 7. DC-Based Vaccines

**Figure 4:**
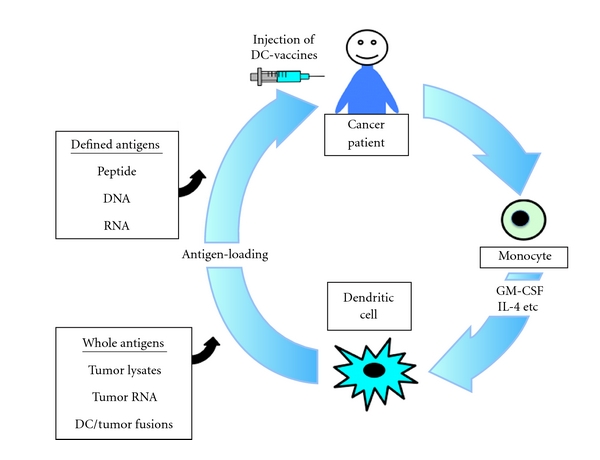
Strategies to deliver defined or whole antigens to DCs. DCs used for cancer vaccines have been generated from the peripheral blood monocytes of the patients using cytokines including GM-CSF and IL-4. To generate antigen-specific CTL response against tumor cells, DCs have been loaded with defined or whole tumor-associated antigens. For example, DCs loaded with synthetic peptide, antigenic DNA, or RNA have been used. Moreover, whole tumor-associated antigens including defined and unidentified have been also loaded to DCs.

DCs derive their potency from the prominent expression of MHC class I and II, costimulatory (CD80 and CD86), and adhesion molecules that provide secondary signals for the activation of naive CD4+ and CD8+ T cells [24]. Therefore, a major area of investigation in cancer immunotherapy involves the design of DCs-based cancer vaccines [71, 72]. Several strategies to deliver TAAs including defined or whole antigens to DCs have been developed to generate a potent CTL response against tumor cells in murine and human systems (Figure 4). DCs have been pulsed with synthetic peptide derived from the known tumor antigens [73], tumor cell lysates [74], apoptotic tumor cells [75], or RMA derived from tumor antigens [76] and transfected with whole tumor cell DNA [77] or RNA [78]. Moreover, DCs have been fused with tumor cells to induce antigen-specific polyclonal CTL responses [79]. In the DC/tumor cell fusion approach, whole TAAs including those known and those yet unidentified are processed endogenously and presented by MHC class I and II pathways in the context of costimulatory signals [80–82]. Although DC-based vaccines have proven clinically safe and efficient to induce tumor-specific immune responses, only a limited number of objective clinical responses have been reported in cancer patients [83–86]. These relatively disappointing results have prompted the evaluation of multiple approaches to improve the efficacy of DC-based vaccines. 

DC-based vaccines have also been used for pancreatic cancer (Table 3). The human tumor antigen mucin, encoded by the gene MUC1, is a high-molecular-weight glycoprotein that is overexpressed in adenocarcinomas including pancreatic cancer and hematological cancers and can be recognized by cytotoxic T lymphocytes (CTLs) and monoclonal antibodies [87]. A vaccine consisting of liposomal MUC1-transfected autologous DCs was evaluated in a clinical phase I/II trial. In MUC1 peptide-loaded DC vaccines in pancreatic and biliary cancer patients following resection of their primary tumors, 4 of the 12 patients followed for over four years were alive, all without evidence of recurrence [88]. Moreover, MUC1-specific immune responses were also observed even in patients with pretreated and advanced disease, following immunization with DCs transfected with MUC1 cDNA [89]. As hTERT is the catalytic subunit of telomerase and a prototype for a novel class of universal tumor antigens, it is one of widely applicable targets recognized by CTLs [90]. In the human system, DCs transfected with hTERT mRNA have previously been shown to induce CTL responses to hTERT in vitro [91]. Furthermore, findings from initial clinical trials demonstrate that hTERT-specific immune responses can be safely induced in cancer patients [92]. A patient who could not continue chemotherapy due to sever neutropenia had been treated with autologous DCs transfected with hTERT mRNA for 3 years and resulted in no evidence of active disease. Moreover, the complete remission (CR) was associated with induction of hTERT-specific CD4+ and CD8+ T cells [93].

## 8. DNA Vaccines

Cell-based cancer vaccines cause antitumor immune response at first. But they become less effective over time because the induced immune system recognizes them as foreign and quickly destroys them. DNA vaccines that consist of TAAs and additional immune-stimulatory factors have an advantage over cell-based vaccines because it can provide prolonged antigen expression, leading to amplification of immune responses and inducing memory responses against weakly immunogenic TAAs. Moreover, as DNA might be taken up by cells and the encoded antigen is processed through both endogenous and exogenous pathways, DNA vaccines that administered via intramuscular injection allow for an immune response to multiple potential epitopes within an antigen to be generated regardless of the patient's MHC profile [95]. DNA vaccines are now being studied in clinical trials for melanoma and prostate cancer. In pancreatic cancer, DNA vaccination targeting MUC1 [96] or survivin [97] has been studied in murine models and resulted in antitumor immune responses.

## 9. Chemotherapy and Immunotherapy

Recently, new paradigms have emerged in the field of cancer vaccine research. In particular, the potential use of combination therapies that incorporate immune modulators and standard radio- and chemotherapy to synergize with cancer vaccines has been discussed. Cytotoxic chemotherapy is generally considered immunosuppressive, because of its toxicity for dividing cells in the bone marrow and peripheral lymphoid tissue. Therefore, the combination of cancer vaccines with chemotherapies has been considered to be inappropriate because the immunosuppressive effects of the chemotherapy would negate the efficacy of cancer vaccines. However, increasing evidences have been mounting to suggest that immunotherapy has the possibility of achieving better success when used in combination with conventional chemotherapy [98, 99]. A standard cytotoxic agent, gemcitabine, not only exerts direct antitumor activity, but also mediates immunological effects relevant for cancer immunotherapy [100–102]. Cross-presentation of TAAs by DCs is essential for induction of augment CTL responses. Treatment of cancer cells and DCs with gemcitabine results in enhanced cross-presentation of TAAs by DCs, CTL expansion, and infiltration of the tumor, all of which are associated with augmented CTL [103–106]. The increase in cross-presentation did not lead to tolerance [104, 105]. Moreover, gemcitabine reduced the number of myeloid suppressor cells but did not reduce CD4+ T cells, CD8+ T cells, NK cells, macrophages, or B cells [107]. Therefore, gemcitabine may be not immunosuppressive and enhance responses to immunotherapy administered to activate or support immune responses directed toward driving effector immunity to pancreatic cancer cells [108]. Indeed, combination of DCs pulsed tumor cells with gemcitabine augmented therapeutic efficacy in vivo in a murine pancreatic cancer model [109]. Moreover, Ramakrishnan et al. [110] have reported that chemotherapeutic agents caused upregulation of cation-independent mannose 6-phosphate receptor (CI-MPR) expression on cancer cells and a concurrent increase in the uptake of granzyme B by activated CTLs in a large number of neighboring cancer cells. As a result, CTLs may cause apoptosis in large numbers of cancer cells manifesting in a clinically evident antitumor effect. Thus, such a combination therapy may be very promising approach to the treatment of patients with advanced pancreatic cancers.

**Figure 5:**
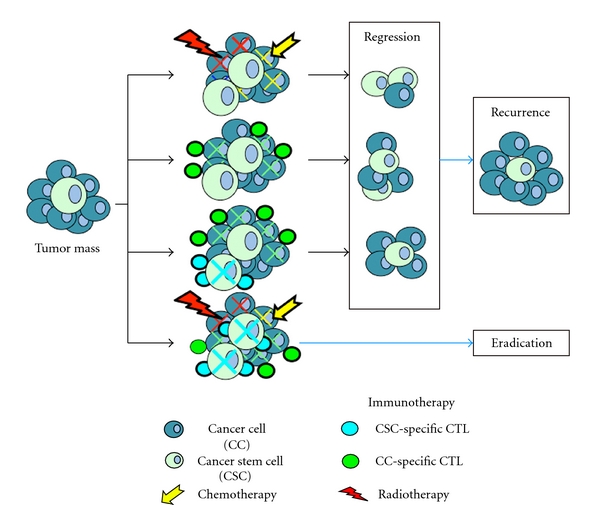
Combination therapies of immunotherapy and standard radio- and chemotherapy. Currently applied standard therapies such as radio- and chemotherapy target bulk cancer cells that are less resistant than cancer stem cells. This leads to initial regression of the tumor mass but eventually regrowth from residual CSCs. Combined therapies of standard therapies and immunotherapeutic approach targeting CSCs would cut off the rejuvenating supply of CSCs and resulted in tumor eradication.

Tumors that develop drug resistance would still be a suitable target for immunotherapy [111]. It has been well known that the majority of patients with advanced pancreatic cancer that respond initially to standard chemotherapies ultimately undergo relapse due to the survival of small populations of cells with cancer-initiating/cancer stem cell (CSC) fraction [112]. These CSCs are a subpopulation of the tumor more capable than other cells to self-propagate, initiate new tumors differentiate into bulk tumor, and therefore sustain tumor growth. Although chemotherapy kills most cancer cells, it is believed to leave CSCs behind, which might be an important mechanism of resistance [113]. CSCs are resistant to a variety of treatments, including chemotherapy and radiotherapy, with varied mechanisms of resistance, including high expression of ATP-binding cassette (ABC) drug transporters, an active DNA-repair capacity, and a resistance to apoptosis [113, 114]. Recently, CSCs have been isolated from various types of malignancies, including pancreatic cancer [114–118]. According to the manner of expression in CSCs, TAAs can be classified into two categories: (i) CSC-specific antigens, such as SOX2 [119] and ALDH1A1 [120] and (ii) shared antigens, such as CEP55 [121], MUC1 [122], or WT1 [123, 124] between CSCs and more differentiated subpopulations. Several methods to isolate CSC have been reported, including cell surface markers such as CD44, CD24, CD133, or epithelial-specific antigen (ESA) and side population (SP) cells using Hoechst 33342 dye [115, 119, 120]. Purified tumor stem cells from a patient can be used to immunize the patient or to activate the donor's immune cells against the tumor stem cells [113]. Therefore, the development of strategies that target the CSC population by immunotherapy may be highly desirable. Success of these potential therapies will depend on how well immunological responses to CSCs can be modulated by vaccines. We recently generated hybrid cells by fusing DCs and CSCs to activate potent CSC-specific CTL responses. The DC/CSC fusions induced proliferation of T cells with high expression levels of IFN-*γ* and enhanced killing of CSCs in vitro [111]. Moreover, peptide-based cancer vaccines or adoptive cell transfer of the CSC-specific CTL clone is a possible approach for targeting chemotherapy-resistant CSCs [120]. These findings open a novel field of investigations for future clinical trial design, taking into account the immunostimulatory capacity of chemotherapy such as gemcitabine, and using them in combined chemoimmunotherapy strategies in patients with pancreatic cancer [103, 104, 106, 125, 126]. Moreover, it seems that a period of time exists between the start of chemotherapy and immunotherapy. As the fact that even without chemotherapy, antitumor immune responses induced by immunotherapy cannot be sustained for a long period of time in patients with cancer. It would be important to establish the optimum timing and scheduling of immunotherapy and chemotherapy, to identify whether this synergistic effect is limited to a specific type of chemotherapy and whether immunotherapy can also augment the clinical effect of chemotherapy [44, 110, 127]. A combined approach of conventional therapies such as radiation or chemotherapy kills the bulk of tumor cells, and CSC-reactive CTL that target CSC fraction may represent a more promising approach for the treatment of patients with advanced pancreatic cancer (Figure 5).

In clinical trials, patients with advanced pancreatic cancer had been treated by combination therapy of standard cytotoxic agent, gemcitabine with personalized peptides [49, 128], or vascular endothelial growth factor receptor 2 (VEGFR2) [50]. The reactive personalized peptides (maximum of 4 kinds of peptides) were administered with gemcitabine to patients with nonresectable pancreatic cancer. Median survival time of all 21 patients was 9.0 months with a one-year survival rate of 38%. Immune boosting in both cellular and humoral responses was well correlated with overall survival. Moreover, in combination therapy of peptide for VEGFR2 with gemcitabine for patients with metastatic and unresectable pancreatic cancer, the median overall survival time of all 18 patients who completed at least one course of the treatment was 8.7 months. VEGFR2-specific CTL responses could be induced by the combination therapy. The survival benefit of combination therapy of peptide vaccines and gemcitabine in comparison with gemcitabine alone needs to be confirmed in randomized clinical trials. Similar findings are also observed in combination therapy of DCs-based cancer vaccines and gemcitabine. Five patients with locally advanced pancreatic cancer had been treated with gemcitabine, OK-432-stimulated DCs injected into the tumor sites, and intravenous infusion of lymphocyte-activated killer cells stimulated with anti-CD3 monoclonal antibody [129]. In this report, 1 patient had partial remission (PR) and 2 had long stable disease (SD) more than 6 months. More recently, we also reported that DC vaccine-based immunotherapies combined with gemcitabine/S-1 were effective in patients with advanced pancreatic cancer refractory to standard chemotherapy [94]. As both WT1 and MUC1 are one of the excellent TAAs for the target of immunotherapy and are frequently expressed in pancreatic cancer cells [36, 37, 123, 130], 38 out of 49 patients had received vaccination with WT1 peptide-pulsed DCs with or without combination of other peptides such as MUC1, CEA, and CA125 in this report. Prior to this combination therapy, 46 out of 49 patients had been treated with chemotherapy, radiotherapy, heavy particle radiotherapy, or hyperthermia but elicited no significant effects. In spite of these handicapped conditions, surprisingly, of 49 patients, 2 patients showed CR, 5 PR, and 10 SD, and median survival time was 360 days. The use of DCs-based vaccines in direct combination with chemotherapy in patients with pancreatic cancer might become a veritable option for the treatment of patients with advanced-stage cancer. Indeed, gemcitabine enhanced WT1 expression in human pancreatic cancer cells and sensitized the pancreatic cancer cells with WT1-specific T cell-mediated antitumor responses [131]. Although the concept is far from being firmly established, these reports may be sufficient to provide a platform for the combination of immunotherapy with chemotherapy. Evaluation is warranted to examine the effect of the approach on disease-free survival and overall survival.

## Figures and Tables

**Table 1:** Peptide vaccines.

Patients	Peptide vaccine	Adjuvant	Response	Ref.
10 resected and 38 advanced pancreatic caner	Mutant K-ras peptide	GM-CSF	Immune response to the peptide vaccine showed prolonged survival compared to nonresponders.	[56]
			K-ras-specific T cells were selectively accumulated in the tumor.	

24 resected pancreatic cancer	Mutant K-ras peptide	GM-CSF	No elicitable immunogenicity and unproven efficacy was observed.	[57]

16 resected or locally advanced pancreatic cancer	100 mer MUC1 peptide	SB-AS2 adjuvant	Detectable MUC1-specific humoral and T-cell responses were detected in some patients.	[59]

6 advanced pancreatic cancer	100 mer MUC1 peptide	Incomplete Freund's adjuvant	One patient showed a tendency for increased circulating anti-MUC1 IgG antibody.	[58]

48 advanced pancreatic cancer	Telomerase peptide	GM-CSF	Immune responses were observed in 24 of 38 evaluable patients.	[60]
			One-year survival for the evaluable patients in the intermediate dose group was 25%.	

11 advanced pancreatic cance	Personalized peptide vaccine		The 6- and 12-month survival rates for patients who received >3 vaccinations (*n* = 10) were 80% and 20%, respectively.	[62]

23 resected pancreatic cancer	Mutant ras long peptide		Seventeen of 20 evaluable patients (85%) responded immunologically to the vaccine.	[65]
			Ten-year survival was 20% (four patients out of 20 evaluable).	

1 liver metastasis of pancreatic cancer refractory to gemcitabine	Survivin peptide		The patient initially underwent partial remission of liver metastasis which proceeded after 6 months into a complete remission with a duration of 8 months.	[40]

**Table 2:** Whole tumor cell-based vaccines.

Patients	Whole tumor cell-based vaccines	Combination	Response	Ref
14 resected pancreatic cancer	Allogeneic GM-CSF-secreting pancreatic cancer cell		Vaccination induced increased delayed-type hypersensitivity (DTH) responses to autologous tumor cells in three patients.	[67]
			3 patients also seemed to have had an increased disease-free survival time, remaining disease-free at least 25 months after diagnosis.	

30 advanced pancreatic cancer	Allogeneic GM-CSF-secreting pancreatic cancer cell	Vaccine alone or in sequence with cyclophosphamide	CD8+ T-cell responses to HLA class I-restricted mesothelin epitopes were identified predominantly in patients treated with cyclophosphamide and immunotherapy.	[70]
			Cyclophosphamide-modulated immunotherapy resulted in median survival in a gemcitabine-resistant population similar to chemotherapy alone.	

**Table 3:** DC-based vaccines.

Patients	DC-based vaccines	Response	Ref
12 pancreatic and biliary cancer patients with resected tumors	MUC1 peptide-loaded DC	4 of the 12 patients followed for over four years were alive.	[88]

10 patients with advanced breast, pancreatic, or papillary cancer	DC transfected with MUC1 cDNA	A vaccine-specific delayed-type hypersensitivity (DTH) reaction was observed in 3 out of 10 patients.	[89]
		4 patients showed a 2- to 10-fold increase in the frequency ofMUC1-specific IFN-gamma-secreting CD8+ T cells.	

1 patient who could not continue chemotherapy due to sever neutropenia	DC transfected with hTERT mRNA	The patient showed no evidence of active disease based on PET/CT scans.	[93]
		The patient developed an immune response against several hTERT-derived Th and CTL epitopes.	

49 patients with advanced pancreatic cancer refractory to standard chemotherapy	Peptide (WT1, MUC1, CEA, and CA125)-loaded DC	2 patients showed complete remission (CR), 5 partial remission (PR) and 10 stable disease (SD).	[94]
	Gemcitabine/S-1	Median survival time was 360 days.	
